# Identifying predictors and measuring variation in hospitalisation for older adult care home residents: a retrospective cohort study using routinely collected data

**DOI:** 10.1093/ageing/afag119

**Published:** 2026-05-06

**Authors:** Carl Marincowitz, Richard Jacques, Katherine Zwerger, Graham Martin, Karen Spilsbury, Jennifer Kirsty Burton, Fawn Harrad-Hyde, David Price, Emily Lam, Hilary M Garrett, Suzanne Mason

**Affiliations:** School of Medicine and Population Health, The Centre for Urgent and Emergency Care Research, The University of Sheffield, Sheffield, England, United Kingdom of Great Britain and Northern Ireland; The University of Sheffield School of Medicine and Population Health, Sheffield Centre for Health and Related Research, Sheffield, England, United Kingdom of Great Britain and Northern Ireland; School of Medicine and Population Health, The Centre for Urgent and Emergency Care Research, The University of Sheffield, Sheffield, England, United Kingdom of Great Britain and Northern Ireland; University of Cambridge - THIS Institute, Cambridge, Cambridgeshire, United Kingdom of Great Britain and Northern Ireland; School of Healthcare, Faculty of Medicine and Health, University of Leeds, Baines Wing, Yorkshire, Leeds LS2 9JT, United Kingdom of Great Britain and Northern Ireland; Academic Geriatric Medicine, School of Cardiovascular & Metabolic Health, University of Glasgow, Room 2.42, New Lister Building, University of Glasgow Glasgow Royal Infirmary, Glasgow, Scotland, G12 8QQ, United Kingdom of Great Britain and Northern Ireland; Health Sciences, Leicester Organisation for the Relief Of Suffering (LOROS), University of Leicester, University Road, Leicestershire, Leicester, LE1 7RH, United Kingdom of Great Britain and Northern Ireland; Optimum Patient Care UK, Cambridge, England, United Kingdom of Great Britain and Northern Ireland; Patient Public Contributor, UK, United Kingdom of Great Britain and Northern Ireland; Patient Public Contributor, UK, United Kingdom of Great Britain and Northern Ireland; The University of Sheffield School of Medicine and Population Health, Sheffield Centre for Health and Related Research, Sheffield, England, United Kingdom of Great Britain and Northern Ireland

**Keywords:** care homes, enhanced health in care homes, inpatient admissions, older people

## Abstract

**Background:**

There over 250 000 care home residents in England and they account for a disproportionate number of hospital admissions. Since 2016, enhanced services for care homes have been developed to reduce admissions but there is regional variation in provision. We used a nationally representative cohort of care home residents to identify predictors of admissions and measure regional risk adjusted variation.

**Methods:**

Using primary care data from a third of practices in England, we derived a cohort of over 40 000 care home residents linked to the national regulator’s register of care homes between 01/01/2023 and 31/12/2024. We estimated a three-level negative binomial regression model including resident, postcode district and commissioner (Integrated Care Board, ICB) level factors which predicted 6-monthly count of admissions. Using this model, we estimated funnel-plots to measure case-mix risk adjusted variation in admission rates at postcode district and commissioner geographical levels.

**Results:**

Significant predictors of admission count included age, gender, frailty, presence of feeding tube, long-term catheterisation, polypharmacy, anticholinergic burden, sedative load, care home rating and proportion of care home jobs filled. Considerable variation in risk adjusted admission rates at commissioner (ICB) level was demonstrated. ICB outliers ranged between having 0.6 and 2.2 times as many admissions as would be expected based on case-mix.

**Conclusion:**

This is one of the largest studies of care home residents in England. We identified potentially modifiable risk factors for admissions and significant variation in ICB level admission rates. This variation could be the result of differences in enhanced services provision and requires further research.

## Key Points

- Previous research has failed to estimate case-mix adjusted variation in hospital admissions for older care residents in the UK.
- We have created one of the largest cohorts of care home residents in England using routinely collected primary care data.
- We have identified important potentially modifiable predictors of likelihood of inpatient admission.
- We found significant commissioner level regional variation in admissions which requires further investigation.

## Background

There were an estimated 278 946 people aged 65 years and over living in a care homes in England and Wales in 2021 [1]. This accounts for around 2·8% of the population aged 65 years and over, yet care home residents account for 8% of emergency admissions in this age group [2]. Significant (20-fold) variation in admission rates for people aged over 75 living in care home-containing postcode areas was demonstrated in England using 2011 census data [3, 4]. Differences in admission rates may reflect differences in residents’ health care needs, provision of care within their care homes or available health services [5].

Despite significant in-depth data collection at care home level [6], there is no nationally collected standardised minimum dataset for care home residents in England equivalent to the minimum datasets collected for residents in Medicare/Medicaid-certified nursing homes in the USA (includes demographics, clinical status measures, physical functioning assessment, psychological status measures and psycho-social functioning measure) [7, 8]. This has limited ‘big data’ studies assessing outcomes for residents. In our recent review, we found no UK studies identifying factors associated with likelihood of transfer to the Emergency Department (ED) from care homes [9]. Several methods have been proposed to identify residents in routinely collected health care data [10, 11]. The Health Foundation cross-referenced patient addresses derived from primary care data with the addresses of care homes registered with the Care Quality Commission (CQC) (care homes must register with the CQC in the UK) to identify all residents in England. Residents were then linked to hospital data to estimate the number of ED attendances and inpatient admissions in 2016–2017 and describe characteristics of residents admitted to hospital [2]. Sherlaw-Johnson *et al*. estimated admissions from care homes in 2011 by using postcode to link admitted residents to care home-containing postcode areas [4].

The limitation of these methods is that since they only identify residents who have been hospitalised, there has been no comparison of hospitalised and non-hospitalised residents. Therefore, factors associated with hospitalisations were not identified or adjusted for. Moreover, although assessing variation in admission rates at care home level is important, since 2016, the National Health Service (NHS) has invested in regional enhanced care services for care homes to reduce hospitalisations of residents [12]. There is regional variation in commissioning, availability and implementation of services [13]. It is therefore also important to measure variation at the level services are commissioned and provided.

We aimed to: (i) derive a cohort of care home residents from routinely collected primary care data, (ii) identify factors associated with likelihood of admission, and (iii) measure case-mix adjusted regional variation in admissions.

## Methods

### Study design

This is a retrospective cohort study using routinely collected data derived from the Optimum Patient Care Research Database (OPCRD) [14]. We adhered to REporting of studies Conducted using Observational Routinely collected health Data Statement guidance [15]. This forms part of a programme of research (UVAC study NIHR303605) [16].

### Setting

In England, health and social care services are commissioned by regional organisations, called Integrated Care Boards (ICBs). At the time of the study, there were 42 ICBs responsible for planning and performance of NHS and social care services. The term ‘care home’ is an umbrella term encompassing settings where people live and receive care and support. This includes care homes without nursing (residential care homes) that provide personal care delivered by care staff, with nursing care provided by community nursing teams, and care homes (nursing homes) which provide personal and 24-hour nursing care. OPCRD provides de-identified routinely collected data for over 1100 UK General Practices (GP) providers (around 35% population coverage). GP providers are organisations that provide primary care to registered patients.

### Data set

#### Primary care data

OPCRD identified residents within their dataset with either READ or SNOMED coding (Appendix 2) indicating they were a care home resident at the beginning (index date) of four 6-monthly periods between 01/01/2023 and 31/12/2024. READ and SNOMED codes are a standardised coded thesaurus of patient findings and procedures [17]. Hospital admissions in the following 6 months were estimated using recording of inpatient discharge letters. This created 4 cohorts with admissions estimated in the 6 months following index date: Cohort 1 (01/01/2023 to 30/06/2023), Cohort 2 (01/07/2023 to 31/12/2023), Cohort 3 (01/01/2024 to 30/06/2024) and Cohort 4 (01/07/2024 to 31/12/2024).

Demographic information was extracted at each index date. Deprivation decile was estimated using postcode of registered GP practice. Cambridge comorbidity index was calculated using all diagnoses recorded prior to index date, as was the electronic frailty index [18, 19]. Polypharmacy, sedative score and anticholinergic cognitive burden scale were estimated based on prescriptions recorded in the 12 months prior to each index date [20–22]. The electronic frailty index, polypharmacy, sedative score and anticholinergic cognitive burden were categorised using cut-offs validated in previous literature and used clinically [20–23]. Based on previous literature, presence of urinary catheter was identified by coding 12 months and feeding tube on coding 24 months, prior to index dates [9].

#### Care quality commission data

The 03/03/2025 CQC register of health and social care organisations in England was downloaded. Duplicate organisations were removed, as were organisations not registered as care homes with services for older people. Care home addresses were then used to cluster care homes by the postcode district (first part of UK postcode, 2302 postcode districts in England and Wales, average population 25 889 inhabitants) [24]. Postcode districts were then clustered by ICBs using Office for National Statics data for ICB coverage (where districts encompassed multiple ICBs they were allocated to the ICB that covered the majority of postcodes within the district) [25]. Using the CQC register, the number and characteristics of care homes within a postcode district were estimated. An estimate of total number of residents per district and ICB was made by aggregating the number of beds per care home in each district and ICB.

#### Data linkage

**Figure 1:**
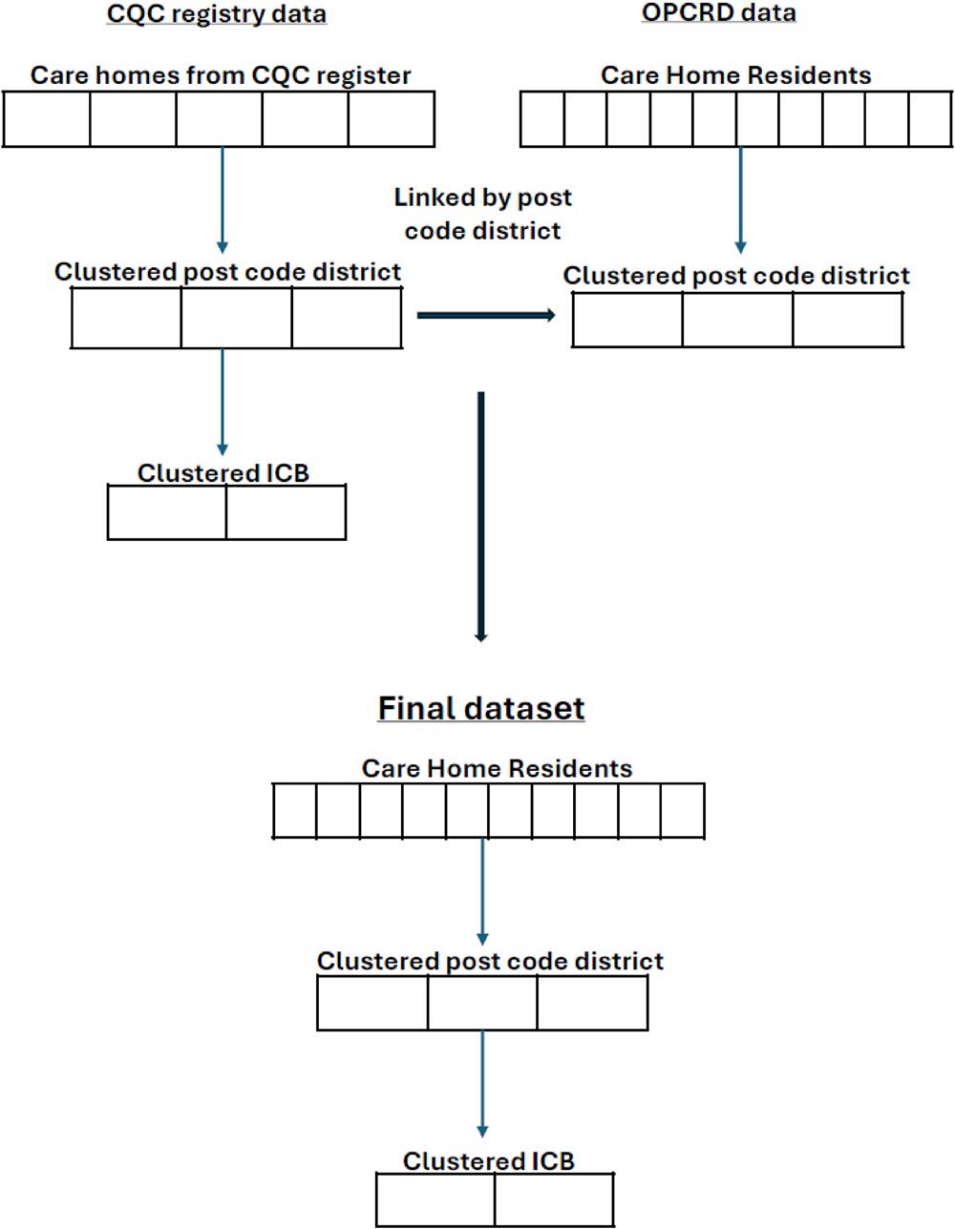
Data linked between CQC register and primary care data sets.

Figure 1 shows how data were linked using postcode district. We excluded postcode districts and ICBs where <10% of care home residents had been sampled (total number of residents estimated by aggregating care home sizes). The Skills for Care adult workforce estimates by ICB were used to estimate percentage of care home jobs filled [26].

### Analysis

Descriptive characteristics of residents, care homes within districts and within ICBs were estimated. Residents with incomplete demographic information were excluded from analysis, as were residents in postcode districts not linkable to the CQC register. A three-level (resident, district and ICB) negative binomial regression model incorporating random intercepts predicting count of inpatient admissions per resident for the first 6-month period was estimated using the STATA menbreg function. Predictors were included based on the findings of three previous systematic reviews, advice from our advisory, and patient and public involvement groups [9, 27, 28]. Fractional polynomials were fitted to test for nonlinear forms for all continuous variables using the STATA mfp function. An offset variable for time in the study was included to account for residents who died or left OPCRD-registered practices within the 6-month analysis period. Ranked caterpillar plots of postcode district and ICB effects with associated 95% confidence intervals were estimated. The analysis was repeated for the following 6-month periods with recalibration of predictor variables.

For each 6-month period, the expected count of admissions per district and ICB based on resident and care home characteristics was estimated using the negative binomial regression model. To identify outliers with higher or lower than expected admissions, funnel plots were produced of the ratio of observed to expected counts plotted against expected counts. The 95% and 99.9% control limits were estimated using a random effects model to allow for overdispersion and we applied a trimming approach (excluding the top and bottom 10% of districts/ICBS that may unduly influence the limits) [29, 30]. Analysis was completed using STATA version 15.1 and R studio.

### Ethics

OPCRD has NHS HRA Research Ethics Committee approval to provide anonymised research datasets for research (NHS HRA REC ref: 20/EM/0148). The Anonymised Data Ethics and Protocol Transparency Committee approved use of the anonymised data for this project (ADEPT0325).

### Patient and public involvement (PPI)

Patients and the public groups have supported study design, particularly advising on inclusion of predictor variables and outcomes. Our Patient and Public Involvement and Experience (PPIE) groups encompassed diverse perspectives, including professionals with experience working in care homes, care home residents, their family and carers.

## Results

### Population characteristics

Appendix 3 shows the derivation of care homes clustered by postcode district and ICB from the CQC register. Appendix 4 shows the derivation of the four 6-monthly care home resident cohorts clustered by districts and ICBs. Cohorts included between 49 174 and 51 155 residents clustered by between 448 and 474 postcode districts, and 32 to 35 ICBs. Table 1 shows the characteristics of the residents, and Table 2 shows postcode district and ICB level characteristics.

**Table 1:** Characteristics of care home resident cohorts.

Population characteristic	Level	01/01/2023–30/06/2023 *n* = 41 480	01/07/2023–31/12/2023 *n* = 43 508	01/01/2023–30/06/2023 *n* = 43 667	01/01/2024–30/06/2024 *n* = 44 705
Age	Median (IQR) Mean (SD)	86 (79–92) 85.1 (8.3)	86 (79–91) 84.7 (8.2)	86 (79–91) 85 (8.3)	86 (79–91) 84.7 (8.2)
Gender	Female	27 792 (67%)	28 982 (66.6%)	28 967 (66.3%)	29 629 (66.3%)
Ethnicity	White (British, Irish other) Asian/Asian British Black/Black British Hispanic Middle Eastern/Middle Eastern British Mixed black/Black British Mixed other Other Unknown/prefer not say	36 377 (87.6%) 305 (0.7%) 148 (0.4%) 116 (0.3%) 29 (0.1%) 81 (0.2%) 57 (0.1%) 142 (0.3%) 4250 (10.2%)	38 073 (87.5%) 332 (0.8%) 158 (0.4%) 126 (0.3%) 31 (0.1%) 70 (0.2%) 65 (0.2%) 144 (0.3%) 4509 (10.4%)	37 829 (86.6%) 345 (0.8%) 176 (0.4%) 132 (0.3%) 32 (0.1%) 72 (0.2%) 69 (0.2%) 146 (0.3%) 4866 (11.1%)	38 140 (85.3%) 337 (0.8%) 169 (0.4%) 126 (0.3%) 32 (0.1%) 67 (0.2%) 74 (0.2%) 143 (0.3%) 5617 (12.6%)
Deprivation index decile	1 (most deprived) 2 3 4 5 6 7 8 9 10 (least deprived)	4036 (9.7%) 6138 (14.8%) 5039 (12.1%) 4495 (10.8%) 3641 (8.8%) 4753 (11.5%) 4162 (10%) 2470 (6%) 4356 (10.5%) 2415 (5.8%)	4512 (10.4%) 6477 (14.9%) 5170 (11.9%) 4537 (10.4%) 4017 (9.2%) 4755 (10.9%) 4262 (9.8%) 2500 (5.8%) 4624 (10.6%) 2654 (6.1%)	4621 (10.6%) 6718 (15.4%) 5386 (12.3%) 4105 (9.4%) 3969 (9.1%) 4839 (11.1%) 3998 (9.2%) 2667 (6.1%) 4792 (11%) 2572 (5.9%)	4628 (10.4%) 6714 (15%) 5460 (12.2) 4283 (9.6) 3984 (8.9%) 5258 (11.8%) 3967 (8.9%) 2694 (6%) 4926 (11%) 2791 (6.2%)
Electronic frailty index	Fit <0.12 Mildly frail <0.24 Moderately frail <0.36 Severely frail	2604 (6.3%) 7857 (18.9%) 12 716 (30.6%) 18 328 (44.2%)	2638 (6.1%) 7887 (18.1%) 13 257 (30.5%) 19 726 (45.3%)	3003 (6.9%) 8218 (18.8%) 13 007 (29.8%) 19 439 (44.5%)	3355 (7.5%) 8394 (18.8%) 13 198 (29.5%) 19 758 (44.2%)
Cambridge multi-morbidity score	Median (IQR) Mean (SD)	3.4 (2.1–4.6) 3.4 (1.9)	3.4 (2.1–4.7) 3.4 (1.9)	3.4 (2.1–4.7) 3.4 (1.9)	3.4 (2–4.7) 3.4 (1.9)
Feeding tube	Present	210 (0.5%)	207 (0.5%)	206 (0.5%)	201 (0.5%)
Long term catheter	Present	891 (2.2%)	918 (2.1%)	892 (2%)	955 (2.1%)
Polypharmacy (prescriptions previous 12 months)	0–4 5–9 10–14 >14	1729 (4.2%) 5545 (13.4%) 9591 (23.1%) 24 640 (59.4%)	1624 (3.7%) 5658 (13%) 9736 (22.4%) 26 490 (60.9%)	1663 (3.8%) 5747 (13.2%) 10,164 (23.3%) 26 093 (59.8%)	1597 (3.6%) 5942 (13.3%) 10,439 (23.4%) 26 727 (59.8%)
Anticholinergic burden scale score (prescriptions previous 12 months)	0 1 2 3 4 >4	14 836 (35.8%) 9043 (21.8%) 5377 (13%) 4370 (10.5%) 2867 (6.9%) 5012 (12.1%)	15 388 (35.4%) 9366 (21.5%) 5586 (12.8%) 4724 (10.9%) 3009 (6.9%) 5435 (12.5%)	15 512 (35.5%) 9505 (21.8%) 5738 (13.1%) 4644 (10.6%) 3064 (7%) 5204 (11.9%)	15 950 (35.7%) 9932 (22.2%) 5716 (12.8%) 4823 (10.8%) 3076 (6.9%) 5208 (11.7%)
Sedative load (prescriptions previous 12 months)	0 1–2 >2	12 467 (30%) 14 630 (35.3%) 14 408 (34.7%)	13 144 (30.2%) 15 133 (34.8%) 15 231 (35%)	13 005 (29.8%) 15 516 (35.5%) 15 146 (34.7%)	13 599 (30.4%) 16 001 (35.8%) 15 105 (33.8%)
Resident-level outcomes (6-monthly)					
Inpatient admission count	Median (IQR) Mean (SD) Range Proportion at least one least admission	0 (0–0) 0.3 (0.6) 0–7 18.6%	0 (0–0) 0.3 (0.6) 0–6 19.4%	0 (0–0) 0.3 (0.6) 0–7 19.2%	0 (0–0) 0.3 (0.6) 0–7 19.1%
Death	Yes	6039 (14.6%)	5527 (12.7%)	5950 (13.6%)	3676 (8.2%)

**Table 2:** Postcode district and ICB characteristics.

District characteristic	Level	01/01/2023–30/06/2023 *n* = 448	01/07/2023–31/12/2023 *n* = 461	01/01/2023–30/06/2023 *n* = 461	01/01/2024–30/06/2024 *n* = 474
District					
Number residents	Median (IQR) Mean (SD)	75 (43–125) 92.6 (71.6)	78 (44–126) 94.4 (73.8)	79 (44–126) 94.7 (74.8)	78.5 (42–127) 94.3 (74.9)
Number care homes	Median (IQR) Mean (SD)	6 (3–9) 6.5 (4.4)	6 (3–9) 6.5 (4.4)	6 (3–9) 6.4 (4.4)	6 (3–9) 6.5 (4.4)
Mean size care home (number of beds)	Median (IQR) Mean (SD)	39 (31.6–47.8) 39.8 (13.1)	39 (31.5–47.7) 39.7 (13.3)	39.6 (31.6–48.3) 40.3 (13.9)	39.3 (31.7–48) 40.2 (13.4)
Proportion total care home bed capacity postcode included in cohort	Median (IQR) Mean (SD)	37% (21.8%–57.9%) 42.9% (30.7%)	37% (22.2%–58.5%) 43.1% (27.6%)	37.3% (22.2%–57.9%) 43.6% (29%)	36.5% (20.5%–56.7%) 42.1% (27.2%)
Percentage care homes nursing	Median (IQR) Mean (SD)	33% (16%–55.6%) 37% (27%)	33% (15%–55.6%) 36% (27%)	33% (15%–50%) 36.4% (27.3%)	33% (16.7%–55.6%) 36.6% (26.9%)
Percentage care homes with dementia care	Median (IQR) Mean (SD)	75% (58%–100%) 72% (24%)	75% (58%–100%) 72% (24%)	75% (60%–100%) 73.6% (23.5%)	75% (60%–100%) 72.7% (23.9%)
Mean care home rating 1: Inadequate 2: Requires improvement 3: Good 4: Outstanding	Median (IQR) Mean (SD)	2.8 (2.7–3) 2.8 (0.28)	2.8 (2.7–3) 2.8 (0.28)	2.8 (2.7–3) 2.8 (0.28)	2.8 (2.7–3) 2.8 (0.27)
Number of ICBs covering district	1 2 3 4	346 (76.8%) 86 (19.2%) 17 (3.8%) 1 (0.2%)	350 (75.9%) 91 (19.7%) 19 (4.1%) 1 (0.2%)	349 (75.7%) 91 (19.7%) 20 (4.3%) 1 (0.2%)	358 (75.5%) 95 (20%) 20 (4.2%) 1 (0.2%)
District-level outcomes (6-monthly)					
Inpatient admissions count	Median (IQR) Mean (SD)	17 (6–34) 22.8 (21.6)	17 (8–35) 24.4 (23)	17 (8–34) 24.1 (22.9)	16 (8–34) 23.9 (23.7)
Deaths number	Median (IQR) Mean (SD)	10 (5–19) 13.5 (12)	9 (4–17) 12 (10.6)	10 (5–18) 12.9 (11.4)	3 (6–11) 7.8 (7.1)
ICB					
ICB characteristic	Level	01/01/2023–30/06/2023 *n* = 32	01/07/2023–31/12/2023 *n* = 33	01/01/2023–30/06/2023 *n* = 34	01/01/2024–30/06/2024 *n* = 35
Number residents	Median (IQR) Mean (SD)	1135.5 (900–1590) 1388 (894.5)	1128 (890–1575) 1403.3 (911.9)	1098 (750–1572) 1354.4 (943.8)	1087 (763–1502) 1348 (907.7)
Proportion total care home bed capacity ICB included in cohort	Median (IQR) Mean (SD)	19.1% (12.6%–22%) 18.2% (5.8%)	20.6% (13.3%–22.6%) 19.2% (6.1%)	19.9% (13.5%–22.6%) 19.5% (6.8%)	20.7% (13.9%–23.9%) 20.2% (7%)
Proportion jobs care home filled	Median (IQR) Mean (SD)	95.8% (94.4%–96.1%) 95.4% (1.6%)	95.8% (94.4%–96.1%) 95.5% (1.6%)	95.8% (94.4%–96.1%) 95.4% (1.4%)	95.8% (94.3%–96.1%) 95.4% (1.7%)
ICB-level outcomes (6-monthly)					
Inpatient admissions count	Median (IQR) Mean (SD)	261.5 (167.5–345.5) 318.8 (247.2)	274 (181–368) 341 (274.1)	270.5 (180–385) 326.9 (253.1)	252 (172–358) 323.6 (237.3)
Deaths	Median (IQR) Mean (SD)	152 (106–232.5) 188.6 (124)	139 (98–213) 167.5 (107.1)	137.5 (98–215) 175 (125.4)	52 (82–125) 105 (74.6)

The mean age of residents across cohorts was 85 years and around 66% of residents were female. There were high rates of frailty (>70% moderately or severely frail electronic frailty index) and polypharmacy (>55% residents more than 14 medications prescribed previous 12 months). On average postcode districts contained 6 care homes, of which 33% were nursing homes, and 75% had dementia care facilities. Around 3.5% of postcode districts contained more than 15 care homes and 6.5% contained 1 care home.

### Predictive factors

The size of statistical association with 6 monthly count of admissions for factors included in the three-level negative binomial model is shown in Table 3. The functional forms of included fractional polynomials are presented in Appendix 5. The ranked district and ICB level effects are presented in Appendix 6.

**Table 3:** Strength of association between factors included in negative binomial regression model and inpatient admission count.

Population characteristic	Unit change	01/01/2023–30/06/2023 IRR (95% C.I.)	01/07/2023–31/12/2023 IRR (95% C.I.)	01/01/2024–30/06/2024 IRR (95% C.I.)	01/07/2024–31/12/2024 IRR (95% C.I.)
Care home resident level					
(Age/10) ^−1^ - 0.1175185344	1 year	5.91e-30 (9.07e-46 to 3.86e-14)	8.27e-41 (3.49e-56 to 1.96e-25)	3.22e-31 (2.93e-46 to 3.55e-16)	1.29e-43 (1.29e-58 to 1.28e-28)
(Age/10) ^−1^× ln (Age/10) -0.2516258932	1 year	1.23e+27 (3.84e+12 to 3.95e+41)	6.94e+37 (5.18e+23 to 9.30e+51)	8.80e+28 (1.34e+15 to 5.77e+42)	1.59e+40 (2.59e+26 to 9.80e+53)
Gender	Male	1.3 (1.2 to 1.4)	1.3 (1.3 to 1.4)	1.3 (1.3 to 1.4)	1.3 (1.3 to 1.4)
Ethnicity	White Asian Black Hispanic Middle Eastern Mixed black Mixed other Other Unknown/prefer not say	Reference 1.01 (0.8 to 1.3) 1.04 (0.7 to 1.5) 0.6 (0.3 to 0.9) 0.99 (0.4 to 2.3) 0.8 (0.4 to 1.3) 1.5 (0.9 to 2.5) 0.9 (0.6 to 1.4) 0.98 (0.9 to 1.1)	Reference 0.9 (0.7 to 1.2) 1.0004 (0.7 to 1.4) 0.9 (0.6 to 1.3) 1.5 (0.7 to 3.2) 1.4 (0.8 to 2.2) 1.1 (0.7 to 1.9) 1.01 (0.7 to 1.5) 0.99 (0.9 to 1.1)	Reference 0.9 (0.7 to 1.1) 0.8 (0.6 to 1.1) 0.7 (0.5 to 1.1) 0.7 (0.2 to 1.8) 0.987 (0.6 to 1.7) 1.2 (0.7 to 2) 0.8 (0.6 to 1.2) 1.05 (0.97 to 1.1)	Reference 0.8 (0.6 to 1.1) 0.8 (0.6 to 1.2) 0.7 (0.5 to 1.1) 0.9 (0.4 to 2.2) 0.3 (0.1 to 0.7) 0.9 (0.4 to 1.3) 0.9 (0.6 to 1.3) 1 (0.9 to 1.1)
Deprivation index decile	1 (most deprived) 2 (least deprived) 3 4 5 6 7 8 9 10	Reference 0.997 (0.9 to 1.2) 0.9 (0.9 to 1.1) 0.96 (0.8 to 1.1) 1.2 (0.97 to 1.4) 0.8 (0.7 to 1.003) 0.9 (0.7 to 1.1) 1.1 (0.9 to 1.3) 1.04 (0.9 to 1.3) 1.1 (0.9 to 1.4)	Reference 0.9 (0.8 to 1.03) 0.8 (0.7 to 0.97) 0.9 (0.8 to 1.04) 1.1 (0.9 to 1.3) 0.8 (0.7 to 0.99) 1.1 (0.9 to 1.3) 1.1 (0.9 to 1.3) 1.1 (0.9 to 1.3) 1.03 (0.8 to 1.3)	Reference 1.004 (0.9 to 1.2) 0.95 (0.8 to 1.1) 1.008 (0.9 to 1.2) 1.2 (1.01 to 1.4) 1.1 (0.9 to 1.3) 1.1 (0.9 to 1.3) 1.5 (1.3 to 1.8) 0.99 (0.8 to 1.2) 1.3 (1.02 to 1.6)	Reference 0.9 (0.8 to 1.01) 0.8 (0.7 to 0.9) 0.97 (0.8 to 1.1) 1.04 (0.9 to 1.2) 0.8 (0.7 to 0.96) 0.8 (0.7 to 1.03) 1.1 (0.95 to 1.4) 0.9 (0.7 to 1.03) 0.8 (0.6 to 0.96)
Electronic frailty index	Fit <0.12 Mildly frail <0.24 Moderately frail <0.36 Severely frail	Reference 1.1 (0.99 to 1.3) 1.2 (1.1 to 1.4) 1.5 (1.3 to 1.7)	Reference 1.1 (1.005 to 1.3) 1.2 (1.1 to 1.4) 1.5 (1.3 to 1.7)	Reference 0.96 (0.9 to 1.1) 0.96 (0.9 to 1.1) 1.2 (1.1 to 1.4)	Reference 0.9 (0.8 to 1.03) 1 (0.9 to 1.1) 1.2 (1.1 to 1.3)
Cambridge multi-morbidity Score	Unit increase	0.99 (0.98 to 1.003)	1.01 (0.99 to 1.02)	0.996 (0.99 to 1.01)	1.004 (0.991 to 1.02)
Feeding tube	Present	1.5 (1.1 to 2)	1.7 (1.3 to 2.2)	1.04 (0.8 to 1.4)	1.3 (0.97 to 1.8)
Long term catheter	Present	1.2 (1.01 to 1.4)	1.3 (1.1 to 1.5)	1.4 (1.3 to 1.7)	1.3 (1.1 to 1.5)
Polypharmacy (prescriptions previous 12 months)	0–4 5–9 10–14 >14	Reference 1.5 (1.3 to 1.8) 1.9 (1.6 to 2.3) 2.7 (2.2 to 3.2)	Reference 1.2 (1 to 1.4) 1.4 (1.2 to 1.6) 2 (1.7 to 2.3)	Reference 1.5 (1.2 to 1.8) 1.9 (1.6 to 2.2) 2.6 (2.2 to 3.1)	Reference 1.3 (1.1 to 1.5) 1.5 (1.3 to 1.8) 2.1 (1.8 to 2.5)
Anticholinergic burden scale score (prescriptions previous 12 months)	0 1 2 3 4 >4	Reference 1.1 (1.02 to 1.2) 1.1 (0.996 to 1.2) 1.2 (1.1 to 1.3) 1.2 (1.1 to 1.3) 1.4 (1.3 to 1.6)	Reference 1.1 (1.01 to 1.1) 1.1 (1.02 to 1.2) 1.1 (1.03 to 1.2) 1.2 (1.1 to 1.4) 1.4 (1.3 to 1.5)	Reference 1.1 (1.002 to 1.1) 1.2 (1.1 to 1.3) 1.1 (1.1 to 1.2) 1.2 (1.1 to 1.4) 1.4 (1.3 to 1.5)	Reference 1.1.(1.1 to 1.2) 1.1 (1.04 to 1.2) 1.2 (1.1 to 1.3) 1.2 (1.1 to 1.3) 1.4 (1.3 to 1.5)
Sedative load (prescriptions previous 12 months)	0 1–2 >2	Reference 0.9 (0.9 to 1.004) 0.9 (0.8 to 0.9)	Reference 1 (0.9 to 1.1) 0.9 (0.8 to 0.9)	Reference 1 (0.9 to 1.01) 0.9 (0.8 to 0.9)	Reference 0.9 (0.9 to 0.98) 0.8 (0.8 to 0.9)
District level					
Number care homes	Care home	0.995 (0.98 to 1.01)	0.99 (0.97 to 1.004)	0.99 (0.98 to 1.01)	0.99 (0.97 to 1.01)
(Percentage care homes nursing+0.0032894611358643)^−2^ −6.956715081	Percentage	0.99 (0.99 to 0.99)	0.999995 (0.99998 to 1.00002)	0.999998 (0.99999 to 1.00001)	0.999994 (0.99999 to 1.000002)
In (percentage care homes nursing+0.0032894611358643) +0.9698536959	Percentage	0.8 (0.7 to 0.9)	0.9 (0.8 to 1.02)	0.9 (0.8 to 1.04)	0.9 (0.7 to 0.99)
Percentage Care homes specialistic dementia	Percentage	1.1 (0.8 to 1.6)	1.2 (0.9 to 1.6)	1.03 (0.8 to 1.4)	1.01 (0.7 to 1.4)
(Mean size care home/100) ^2^–0.167978599	Care home bed	1.9 (0.99 to 3.5)	1.1 (0.6 to 2)	1.7 (0.9 to 3)	1.6 (0.8 to 3.2)
(Mean care home rating) ^2^–7.934879518	Mean CQC rating	1.8 (1.1 to 2.7)	2.1 (1.3 to 3.2)	2.3 (1.5 to 3.5)	1.5 (0.99 to 2.4)
(Mean care home rating) ^3^–22.3516972	Mean CQC rating	0.9 (0.8 to 0.96)	0.9 (0.7 to 0.9)	0.8 (0.7 to 0.9)	0.9 (0.8 to 0.99)
Number ICBs district in	1 2 3 4	Reference 0.96 (0.8 to 1.1) 0.8 (0.5 to 1) 0.1 (0.1 to 0.3)	Reference 0.9 (0.8 to 1.1) 0.9 (0.7 to 1.2) 0.1 (0.01 to 0.3)	Reference 0.95 (0.8 to 1.1) 1.04 (0.8 to 1.4) 0.1 (0.01 to 0.4)	Reference 1.003 (0.8 to 1.2) 1.2 (0.9 to 1.7) 0.04 (0.01 to 0.3)
ICB level					
(Proportion jobs care home filled×10)^3^ –860.3173152	Percentage	0.6 (0.4 to 0.8)	0.5 (0.4 to 0.7)	0.7 (0.4 to 1.4)	0.6 (0.4 to 0.9)
(Proportion jobs care home filled×10) ^3^× ln (Proportion jobs care home filled×10) –1937.807769	Percentage	1.2 (1.1 to 1.4)	1.3 (1.1 to 1.5)	1.1 (0.9 to 1.5)	1.2 (1.1 to 1.5)

Important predictors of admission count across cohorts included age, male gender, frailty, presence of feeding tube, presence of catheter, polypharmacy, anticholinergic burden, sedative load, average care home rating and proportion of care home jobs filled. Increasing age was associated with increased likelihood of hospital admission until an age of 80 when the relationship reversed (Appendix 5). While increasing frailty, polypharmacy and anticholinergic burden were associated with increased likelihood of hospital admission, increasing sedative load was associated with reduced likelihood. An average CQC rating of more than 3 (good) for care homes present in a district was associated with reduced likelihood of admissions (Appendix 5).

### Observed versus expected admission rates ICB level

**Figure 2:**
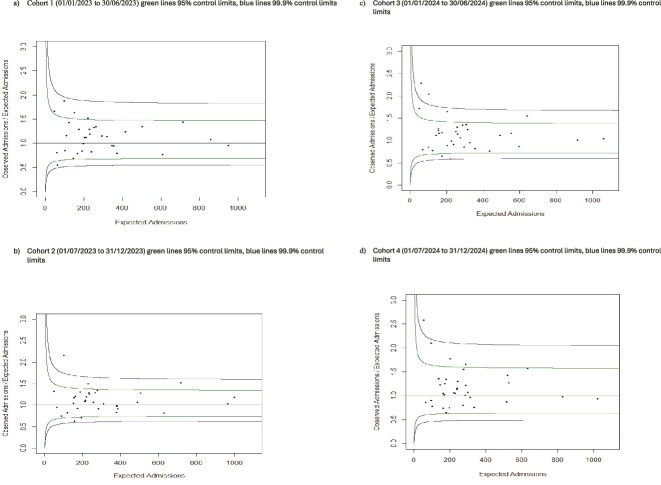
ICB level funnel plots of observed versus expected inpatient admissions care home residents.

Figure 2 shows the funnel plots estimated of the ratio of observed to expected admissions at ICB level. Equivalent funnel plots estimated for districts are shown in Appendix 7.

Considerable variation at ICB level in expected compared to observed admissions was found. The same ICB was an outlier at a 95% confidence interval in all four 6-monthly periods having between 1.9 and 2.2 times as many admissions than would be expected. Conversely, another was an ICB outlier at 95% confidence intervals in two 6-monthly periods had between 0.6 and 0.7 times as many admissions than expected. We found even more variation at postcode district level. However, due to the larger number of included districts with small numbers of residents sampled in some districts and correspondingly small numbers of observed inpatient admission counts, these results need to be interpreted more cautiously. An outlying district at a 99% confidence interval in two 6-monthly periods with over 100 residents sampled in each period (around 20% of residents in district based on the number of care home beds) had between 3.8 and 4.2 times as many observed admissions than would be expected. Conversely, a district with around 75 residents sampled in each 6-monthly period (50% of total residents) was an outlier at a 99% confidence interval in all four 6-monthly periods with between 0.06 and 0.2 times as many admissions than expected.

## Discussion

### Summary

We have created four 6-monthly cohorts of ~20% of care home residents in England [24]. Our modelling identified predictors of admission count, some of which, like polypharmacy and anticholinergic burden, are potentially modifiable. We compared observed admissions to those predicted based on case-mix at district and ICB levels, allowing case-mix adjusted regional variation in admissions from care homes to be measured for the first time in England. We demonstrated significant case-mix adjusted variation at both ICB and postcode district level.

### Comparison to previous literature

Our sample appears representative of the care home population when compared with 2021 census data for England. In the census, the median age of residents was 86 years, around 66% of residents were female, and 97.5% of the population was white [1]. Only a third of care homes were nursing homes but 49% of residents lived in nursing homes. Across our cohorts the median age was 86, around 67% were female, 87.6% were white (over 10% were unknown) and 33% of care homes were nursing homes. We were unable to determine if residents resided in residential or nursing homes in our data.

The Health Foundation previously estimated residents are admitted to hospital 0.7 times per year in England, similar to our mean estimate of 0.3 admissions per 6-month period [3]. Sherlaw-Johnson estimated care homes had a crude annual rate of 0.52 admissions per bed in 2011 with outlying care home containing postcode areas (a smaller geographical unit than post code district) having between 2 to 4 times and less than 0.2 times as many admissions than would be expected based on the number of care home beds (10- to 20-fold variation) [3]. Although not case-mix adjusted, this is a similar degree of variation to that identified at postcode district level. As with our analysis, they also identified that a small proportion of residents accounted for most admissions, with a small number having >10 admissions in a year.

The predictive factors identified are consistent with previous literature (although most research has been conducted in North America or Australia). As in our study, gender, age, polypharmacy, frailty, catheterisation and presence of feeding tubes have been identified as predicting hospitalisations across multiple studies in previous systematic reviews [9, 22, 31–33]. The nonlinear relationship between age and likelihood of admission has also been previously identified [9]. A systematic review found comorbidity did not consistently predict likelihood of admission for residents across included studied [9]. We found no association between comorbidity and likelihood of admission. At an organisational level, as with our study, staffing and care home rating have been found to be predictive of hospitalisations of residents [9, 32].

### Strengths and limitations

This is one the largest cohort studies of care home residents in England. The use of primary care data linked to the CQC register allowed us to assess the predictive value of individual and organisational factors in combination.

We are making inferences at a population level using a 20% sample of care home residents in England. The validity of these inferences depends on how representative our sample is of the national care home population and of residents in included postcode districts and ICBs. Our sample does appear representative of the national population when compared to census data, but there may be differences between residents registered with GP practices which submit to OPCRD and other residents. Comparison of methods for identifying care home residency in routine data found that identifying residents using READ and SNOMED coding may underestimate the number of residents compared with identification using linkage of address [10]. Due to anonymisation, it was not possible for us to use address. However, as part of the Enhanced Health in Care Home Framework all care homes in England should now have a named primary care practice, increasing identification of residency using READ and SNOMED coding in primary care data. The use of coding of inpatient discharge letters may also mean admissions are underestimated. However, sensitivity analysis comparing data submitted by GP practices known to have accurate coding of discharge summaries and other practices found no significant difference in number of inpatient admissions recorded.

The factors included in our predictive model and the accuracy of their measurement are limited by routine collection in primary care data and the CQC register. Demographics, apart from ethnicity and predictors based on prescription information (due to use of electronic prescribing), are likely to be well recorded and accurate. However, individual coding of disease and comorbidities has been found to be subject to random error. The use of the Cambridge comorbidity scale and electronic frailty index, which combines multiple individual diagnostic codes to predict patient outcomes, has been validated in UK primary care datasets for use in research [19]. We were unable to differentiate between residents living in care homes with or without nursing care at an individual level in the available data (although conducted some adjustment for this using characteristics of care homes at district level). There is evidence that enhanced care may have a greater effect in reducing emergency hospitalisations from care homes without nursing care [34]. The available data also did not include any information regarding advance care planning or reason for hospital admissions (e.g. hypoglycaemia). The CQC register contains information based on the point of registration and last inspection so may contain information which is no longer applicable.

### Implications

The associations between frailty, polypharmacy, urinary catheterisation, presence of feeding tubes and anticholinergic load and increased risk of admission are important because they represent potentially modifiable factors where interventions can be targeted. They are identifiable using routinely collected data and can inform personalised care plans formed as part of the Enhanced Health in Care Homes programme [12]. The case-mix adjusted variation in admission rate identified at ICB and postcode district level indicates that there may be variation in commissioned and provided services which may contribute to the observed variation. Although nationally mandated, the Enhanced Health in Care Homes programme provides a broad set of recommendations aimed at reducing the need for treatment in hospital. There are differences in how services are commissioned at ICB level and provided at district level [13]. This analysis is the first part of a programme of research assessing whether variation in admissions may be caused by differences in implementation of the Enhanced Health in Care Home programme. To build on the results reported here, we will undertake in-depth case study analysis of differences in enhanced services in districts embedded in ICB outliers with high, average and low risk-adjusted admission rates [16].

Although needing further validation and refinement (our model incorporated many predictors which may need to be reduced for use in smaller datasets), the modelling method could have been used to estimate case-mix adjusted variation at care home level if our dataset had included individual addresses. A national dataset of care home residents in England linkable to routinely collected health care data is required to make the analysis conducted more robust and applicable to the whole country. Such data needs to be able to distinguish between residents of care homes which provide nursing care and care homes which do not to assess whether programmes such as the Enhanced Health in Care Homes programme affect these populations differently.

## Conclusion

We have conducted one of the largest cohort studies of care home residents in England. We have identified important risk factors for admissions that can be potentially targeted by interventions and significant variation in risk-adjusted admission rates at commissioner and provider level. Our future research will investigate whether this observed variation reflects differences in provision of enhanced care services and the experiences of people living and working in care homes.

## Supplementary Material

aa-25-3209-File005_afag119

aa-25-3209-File004_afag119

## Research data Transparency and Availability

Data may be obtained from a third party and are not publicly available. The data used for this study are subject to data sharing agreement with Optimum Patient Care which prohibits further sharing of individual level data. The datasets used are obtainable from these organisations subject to necessary authorisations and approvals.
