# Supplementary material for: Identifying predictors and measuring variation in hospitalisation for older adult care home residents: a retrospective cohort study using routinely collected data
Source: Age Ageing. 2026 May 6;55(5):afag119. doi: 10.1093/ageing/afag119 (PMC13147443; doi:10.1093/ageing/afag119)
Supplement: aa-25-3209-File005_afag119 [file aa-25-3209-file005_afag119.docx]

**Identifying predictors and measuring variation in hospitalisation for older adult care home residents: a retrospective cohort study using routinely collected data**

Appendix 1: Codes used to identify care home residents

Appendix 2: Derivation of care homes clustered by postcode district and ICB from the CQC register

Appendix 3: Derivation of the four 6-monthly care home resident cohorts clustered by districts and ICBs

Appendix 4: Functional forms of included fractional polynomials

Appendix 5: Ranked district and ICB level effects

Appendix 6: Funnel plots estimated for districts

Appendix 1: Codes used to identify care home residents

Appendix 2: Derivation of care homes clustered by postcode district and ICB from the CQC register

Grouped ICB

*N = 42*

Grouped Postcode District

*N = 1860*

Full CQC register

*N* = 55,293

*N=206*

*Duplicate not primary ID*

*N = 55,087*

Registered care home with services for older adults

*N= 10,863*

Final number care homes

*N = 10,659*

*N* = *44,224*

*Not registered care home or no services for older people*

*Duplicate postcode and first line address based on most recent date of rating*

*N = 204*

Appendix 3: Derivation of the four 6-monthly care home resident cohorts clustered by districts and ICBs

**01/01/2023- 30/06/2023**

Patients with care home resident flag

*N* = 49,174

*N=551*

*Missing sex or deprivation index*

*N = 48,623*

*N* = *1,282*

*Post code district not linkable to CQC register*

*N= 47,341*

*N = 5,836*

*Residents in districts or ICBs with less than 10% care home bed capacity sampled*

*N = 41,505*

*N = 25*

*Residents in districts where 1 or more care homes yet to be assigned CQC rating*

*N = 41,480*

Clustered by 448/1860 (24.1%) care home containing post code districts and 32/42 ICBs in England.

**01/07/2023- 31/12/2023**

Patients with care home resident flag

*N* = 51,097

*N=580*

*Missing sex or deprivation index*

*N = 50,517*

*N* = *1,704*

*Post code district not linkable to CQC register*

*N= 48,813*

*N = 5,271*

*Residents in districts or ICBs with less than 10% care home bed capacity sampled*

*N = 43,542*

*N = 34*

*Residents in districts where 1 or more care homes yet to be assigned CQC rating*

*N = 43,508*

Clustered by 461/1860 (24.8%) care home containing post code districts and 33/42 ICBs in England.

**01/01/2024- 30/06/2024**

Patients with care home resident flag

*N* = 51,155

*N=587*

*Missing sex or deprivation index*

*N = 50,568*

*N* = *1,801*

*Post code district not linkable to CQC register*

*N= 48,767*

*N = 5,051*

*Residents in districts or ICBs with less than 10% care home bed capacity sampled*

*N = 43,716*

*N = 49*

*Residents in districts where 1 or more care homes yet to be assigned CQC rating*

*N = 43,667*

Clustered by 461/1860 (24.8%) care home containing post code districts and 34/42 ICBs in England.

**01/07/2024- 31/12/2024**

Patients with care home resident flag

*N* = 50,877

*N=594*

*Missing sex or deprivation index*

*N = 50,283*

*N* = *1,857*

*Post code district not linkable to CQC register*

*N= 48,426*

*N = 3,671*

*Residents in districts or ICBs with less than 10% care home bed capacity sampled*

*N = 44,755*

*N = 50*

*Residents in districts where 1 or more care homes yet to be assigned CQC rating*

*N = 44,705*

Clustered by 474/1860 (25.5%) care home containing post code districts and 35/42 ICBs in England.

Appendix 4: Functional forms of included fractional polynomials

Age

Proportion care homes nursing district

Mean size care home district

Mean CQC rating care home in a district

Proportion care home jobs filled in an ICB

Appendix 5: Ranked district and ICB level effects

1. 01/01/2023- 30/06/2023

1. 01/07/2023- 31/12/2023

1. 01/01/2024- 30/06/2024

1. 01/07/2024- 31/12/2024

Appendix 6: Funnel plots estimated for districts

1.
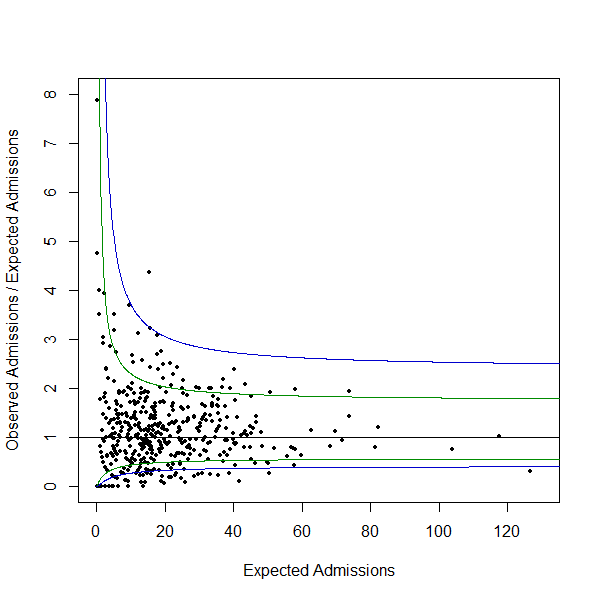
01/01/2023- 30/06/2023
2. 01/07/2023- 31/12/2023


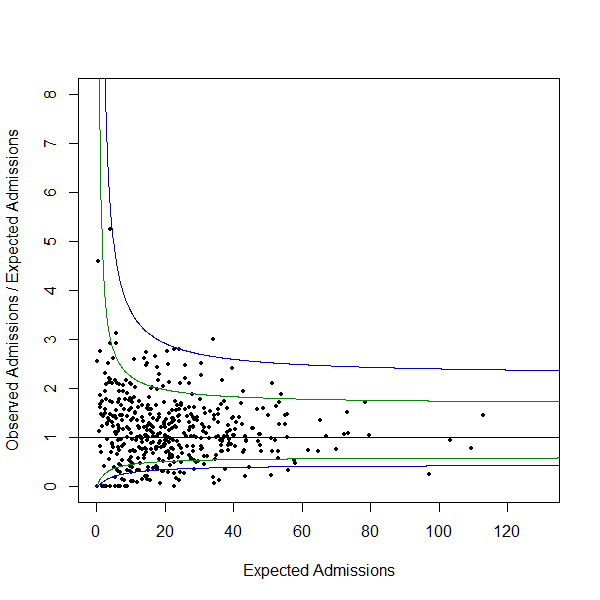


1. 01/01/2024- 30/06/2024


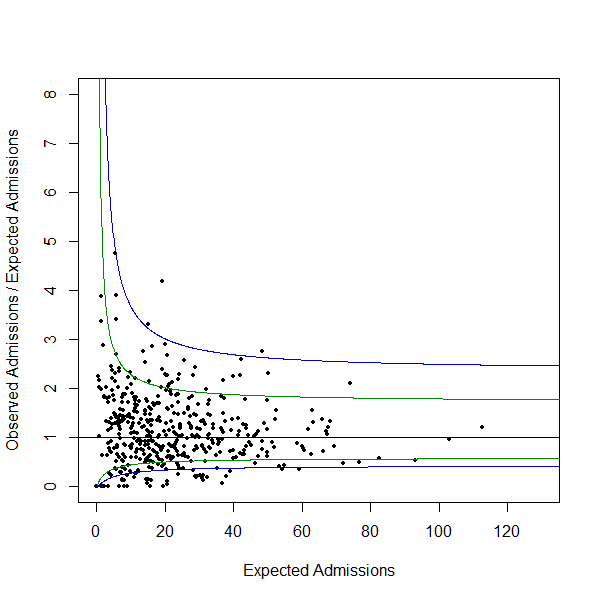


1. 01/07/2024- 31/12/2024

**
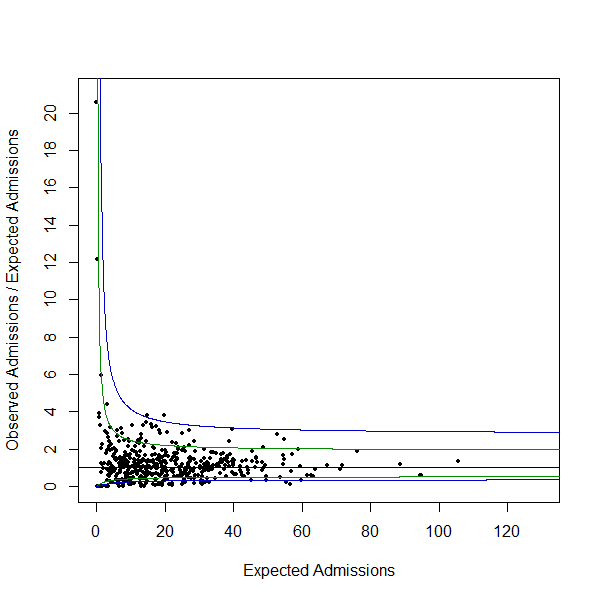
**
